# Metastasis of Malignant Melanoma to Urinary Bladder: A Case Report and Review of the Literature

**DOI:** 10.1155/2015/173870

**Published:** 2015-05-28

**Authors:** Rashna Meunier, Gyan Pareek, Ali Amin

**Affiliations:** ^1^Department of Pathology and Laboratory Medicine, Rhode Island Hospital, The Warren Alpert Medical School, Brown University, Providence, RI 02903, USA; ^2^University Urological Associates, The Warren Alpert Medical School, Brown University, Providence, RI 02904, USA

## Abstract

*Aims*. Metastatic malignant melanoma of the urinary bladder is a rare clinical entity, with only twenty-three published cases to date. We present a case of this rare entity, a thorough review of the literature, and differential diagnosis of melanoma in the bladder. *Methods and Results*. A 55-year-old woman with a history of malignant melanoma of the right thigh, excised eight years ago, presented with back pain, fatigue, and hematuria. She underwent computed tomography (CT) scan and was found to have metastases within the liver, spleen, lungs, and urinary bladder. She underwent cystoscopy and transurethral resection of three polypoid lesions. Histologic and immunohistochemical examination revealed metastatic malignant melanoma involving bladder mucosa. *Conclusions*. This case illustrates the importance of including malignant melanoma in the differential diagnosis of high grade neoplasms of bladder, especially in cases where the relevant clinical history is not available.

## 1. Introduction

Metastasis of malignant melanoma to the bladder is extremely rare in clinical practice. Although many recent review papers report less than ten cases in the past thirty years, our thorough review of the current literature reveals 23 confirmed cases published to date. This indicates that the incidence is likely higher than previously thought and may be due to the fact that many patients are asymptomatic and only found at autopsy. In 1964, Das Gupta and Brasfield performed an autopsy series of 125 patients with metastatic melanoma and found that 18% had metastases to the bladder [1]. Similarly in 2000, Bates and Baithun described five postmortem cases of melanoma metastasizing to the urinary bladder among 153 autopsy cases [2]. Symptomatic patients, on the other hand, usually present with painless gross hematuria [3]. The most common sites of metastasis include regional lymph nodes, lungs, liver, and brain. Malignant melanoma is extremely diverse in its histologic, cytological, and architectural appearances and is often known as the “great mimicker.” This holds true for metastases to the urinary bladder. Our case highlights the importance of including malignant melanoma in the differential diagnosis of high grade urothelial lesions. Although a clinical history of melanoma is often the first clue to the diagnosis, this clinical information is not always readily available.

## 2. Materials and Methods

**Figure 1:**
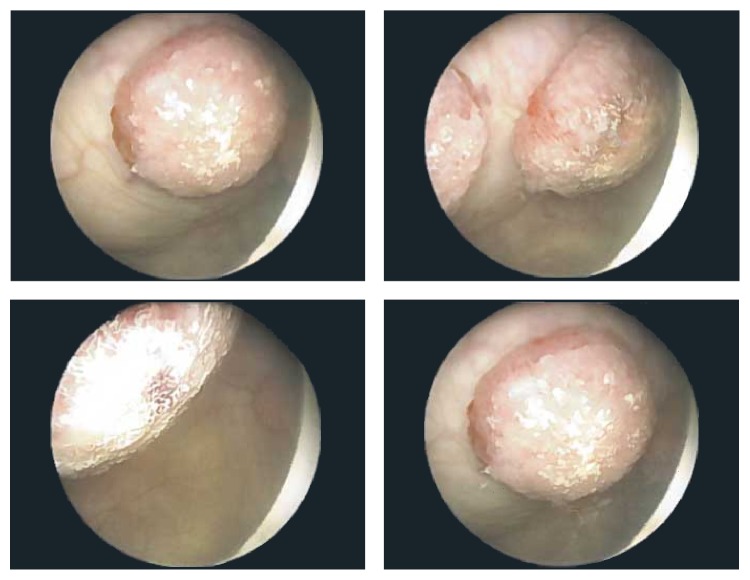
Cystoscopy revealed three polypoid masses, each protruding from a stalk.

A 55-year-old woman presented with back pain, generalized fatigue, and hematuria. Her past medical history was significant for malignant melanoma of the right thigh, diagnosed eight years ago, for which she underwent wide local excision and sentinel lymph node biopsy. Biopsy revealed that the melanoma was 1.05 mm in thickness, Clark's level IV, and without ulceration. One sentinel lymph node was removed which was negative for metastatic disease. Reexcision revealed the presence of scar with no evidence of residual disease. She was followed up regularly and remained asymptomatic, until eight years later when she developed back pain and generalized fatigue. She was evaluated by her primary care physician and tested negative for Lyme disease. Shortly afterwards, she developed hematuria and was referred to urology for further evaluation. Urinalysis ruled out infection and confirmed the presence of hematuria. She also had computed tomography (CT) scan of the chest, abdomen, and pelvis, which revealed multiple liver and splenic lesions, multiple subcentimeter bilateral pulmonary nodules suspicious for metastatic disease, and several masses within the bladder, measuring up to 1.9 cm in greatest dimension. Urine cytology identified atypical cells, but evaluation was limited by scant cellularity. She underwent cystoscopy, which revealed three polypoid, spherical lesions, each protruding from a stalk (Figure 1). Two of these lesions were located along the left anterior bladder dome and measured approximately 2 cm each. A third smaller lesion, located along the left lateral bladder wall, measured approximately 1 cm. There was no evidence of discoloration in the lesions or elsewhere in the bladder. Monopolar loop electrocautery was used to excise the tumors and the bases of the lesions were fulgurated.

## 3. Results

**Figure 2:**
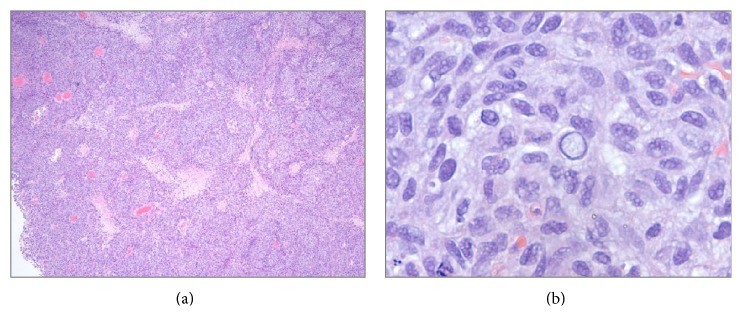
(a) Sheets and nests of tumor cells interspersed with patchy areas of necrosis (hematoxylin-eosin, 40x). (b) The tumor cells have a moderate degree of nuclear pleomorphism and occasionally demonstrate intranuclear and cytoplasmic pseudoinclusions (hematoxylin-eosin, 400x).

Histologic examination revealed diffuse hypercellular sheets and nests of basophilic medium to large tumor cells with high nuclear to cytoplasmic ratio, moderate nuclear pleomorphism, irregular nuclear contours, abundant intranuclear and cytoplasmic pseudoinclusions, vesicular chromatin, and occasional prominent nucleoli (Figure 2). Brisk mitotic activity was noted with atypical mitotic figures. Tumor cells were noted to engulf other tumor cells (tumor cannibalism). Numerous apoptotic bodies and karyorrhectic debris were present, along with patchy areas of geographic tumor necrosis with tumor cells palisading around necrotic foci. The sheets of tumor cells invaded into the muscularis propria of the bladder wall. There was no evidence of intracytoplasmic melanin pigmentation or pigment incontinence. Due to surface ulceration, no evidence of superficial urothelium was identified. Biopsy of the patient's previous skin lesion was not available for comparative review.

**Figure 3:**
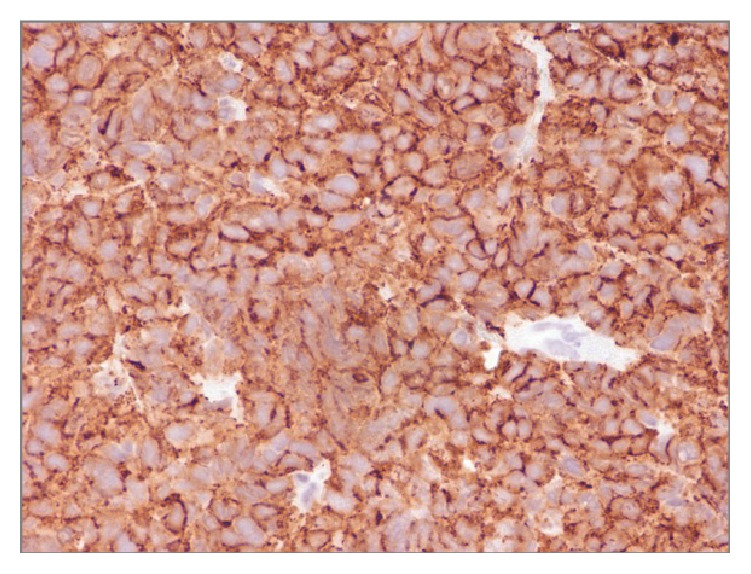
Tumor cells are strongly and diffusely positive for Melan-A.

Immunohistochemistry (IHC) of the bladder lesion revealed diffuse positivity with Melan-A (Cell Marque, Rocklin, CA) and S100 protein (Dako, Carpinteria, CA) (Figure 3). Tumor cells did not stain with p63 (BioGenex Laboratories, San Ramon, CA) or cytokeratin 34BE12 (Enzo Life Sciences, New York, NY). A final histopathological diagnosis of metastatic malignant melanoma involving bladder mucosa was rendered.

## 4. Discussion

The presence of metastatic malignant melanoma within the urinary bladder is an exceedingly rare finding. A literature review by Efesoy and Cayan in 2011 states that there are less than ten cases reported in the last 30 years in the English literature [4]. Similarly, in 2014, Rishi et al. stated that there are “less than score of cases” [5]. This number is cited as slightly higher in a case report by Paterson et al. in which fourteen cases are listed [3]. We conducted a thorough PUBMED search and found that there are a total of twenty-three reported cases (Table 1). Patient age ranged from 28 to 84 years with a mean age of 56.5 years. Men were more commonly affected than women (M : F = 15 : 8). Not surprisingly, the most common primary site of disease was the skin. Sixteen cases had synchronous metastases of melanoma in other sites, five of which were considered “widespread.” The most common presenting symptom in bladder metastasis was hematuria, present in sixteen cases.

While the most common imaging modality for bladder tumors is magnetic resonance imaging, cystoscopy is useful in its ability to visualize the bladder mucosa. However, there are cases in which the mucosa is uninvolved and the metastatic melanoma only involves the bladder wall [6]. Of all the cases published to date, synchronous metastases were present in all but four patients. Unfortunately, the long term followup for the patients without synchronous metastases was not available for comparison with those having synchronous disease.

In cases where the clinical history of primary melanoma is noted, the diagnosis of bladder metastases is often straightforward and can be confirmed by immunohistochemical stains. However, in the absence of a clinical history of melanoma, other lesions with an infiltrative growth pattern and similar cytomorphology may be considered. The possible list of differential diagnoses includes high grade urothelial carcinoma, prostatic carcinoma in male, Müllerian carcinomas in female, lung carcinoma, breast carcinoma, and rare entities like paraganglioma and sarcomatoid carcinoma.

When faced with this diagnostic challenge, there are certain morphological features that can be helpful in differentiating metastatic melanoma from high grade urothelial carcinoma. Malignant melanoma usually presents as a nodule devoid of mucosal excrescences and papillary fronds. The overlying urothelium is mostly unremarkable without any in situ components. In general, the absence of a superficial urothelial lesion overlying a poorly differentiated tumor is an important clue to secondary bladder neoplasia, including metastatic melanoma [5]. The presence of intramucosal atypical melanocytes has been reported in primary bladder melanomas and is considered a prerequisite finding for the diagnosis of primary bladder origin [7]; however, there is no report of such lesions in secondary bladder melanoma.

Tumor cells in melanoma are predominantly comprised of epithelioid cells with rare spindle cell morphology. They reveal macronuclei with marked variation in nuclear size and shape. Nuclei are hyperchromatic with prominent large eosinophilic nucleoli. A helpful feature is the conspicuous presence of intranuclear inclusions. The stroma surrounding melanoma is usually desmoplastic and inflamed, with melanophage aggregation. Intracytoplasmic pigments are useful when present. Atypical mitoses and lymphovascular invasion can also point to secondary bladder tumors including melanoma. It is noteworthy that none of the above-mentioned morphological findings are pathognomonic and a multimodal approach should be considered when confronting a suspicious secondary bladder tumor. The diagnosis can be confirmed using a panel of IHC (vide infra).

Urothelial carcinoma on the other hand is usually associated with a papillary or flat carcinoma in situ (CIS) component on the surface of the lesion that is continuous with the invasive component. Urothelial lesions commonly show glandular or squamous differentiation without cytoplasmic pigmentation or pigment incontinence around tumor nests. IHC staining for high molecular weight cytokeratin (34BE12 and CK5/6), p63, uroplakin, thrombomodulin, and GATA3 supports urothelial origin.

Prostate adenocarcinoma in males lacks cellular pleomorphism. Tumor cells are typically uniform and form glandular and cribriform arrangements. There is generally a high tendency for invasion into perineural spaces in the tumor. IHC staining for prostate-specific antigen (PSA), prostate-specific membrane antigen (PSMA), alpha methylacyl-CoA racemase (AMACR), and prostein (P501s) can be very helpful in differential diagnosis.

Metastatic Müllerian tumors are diverse and, based on the subtype and organ of origin, can show variable morphologies. Some variants like high grade serous carcinoma can show marked cellular pleomorphism and extension beyond the organ of origin. Such tumors show micropapillary clusters with psammomatous calcifications. These tumors are usually cytokeratin positive and based on the site of origin can express PAX8, estrogen (ER), and progesterone (PR) receptors.

Breast carcinoma can be seen metastasizing into bladder and other pelvic organs. Arrangement of tumor cells in single file, discohesive tumor cells, and intracytoplasmic vacuoles can often be seen in metastasizing lobular breast carcinoma. However, some other carcinomas like lung may not show any specific morphological features to suggest a secondary tumor. In such situations, IHC is very helpful in the differential diagnosis. In the case of breast cancer, ER, PR, GATA3, and mammaglobin can be helpful, while TTF1 and napsin-A can point toward lung adenocarcinoma.

Paraganglioma of the bladder can also mimic melanoma. Occasionally these tumors cause prominent clinical symptoms. Morphologically, bladder paraganglioma is composed of a monomorphic cell population with or without cytoplasmic clearing and rare mitosis without atypical forms. IHC for chromogranin-A and synaptophysin is helpful in the diagnosis [8].

Rishi et al. suggest that metastatic melanoma often has nonrefractile green-brown pigment on gross examination [5]. In the available literature, only five case reports have noted the presence of grossly pigmented dark blue or black lesions on cystoscopy [3, 9–11]. The current case did not show any evidence of discoloration within the lesion. The histologic presence of pigment within lesional cells or surrounding histiocytes should prompt the pathologist to consider metastatic melanoma in the differential diagnosis.

Melanosis, both with and without atypia, has been reported in about a dozen cases in the literature [12]. Although melanosis usually occurs independently without any associated or adjacent lesions, it may be seen with other malignancies. One must keep in mind that the presence of melanosis within the bladder does not necessarily confer the diagnosis of melanoma. There are two reported cases of melanosis with subsequent development of urothelial carcinoma and one reported case of melanosis with subsequent development of multifocal malignant melanoma [13–15].

Sarcomatoid carcinoma may mimic metastatic melanoma with spindle cell morphology. The cells of sarcomatoid carcinoma are often large, spindled, and pleomorphic and may appear similar to the pleomorphic melanocytes of melanoma. However, the presence of heterologous elements such as cartilage or bone should point towards a diagnosis of sarcomatoid carcinoma. To confirm the diagnosis, areas of the tumor with epithelial differentiation should be cytokeratin positive, whereas melanoma will be negative.

To confirm the diagnosis of metastatic malignant melanoma, IHC is invaluable. S100 protein is a sensitive but not very specific marker and should be confirmed by more specific markers including Melan-A, HMB45, and MITF. One must be aware of the variable expression of melanoma markers in metastatic melanoma. The heterogeneity of melanoma associated antigens can even be seen within a single lesion. Therefore, we recommend the use of more than one melanoma marker. Cytokeratin expression, on the other hand, has been reported in up to 2% of melanomas [16]; therefore, this marker can be reliably used to rule out other carcinomas.

When confronting a malignant melanoma of the bladder in the absence of any clinical evidence of a primary lesion, one must keep primary malignant melanoma of the bladder within the differential diagnosis. Primary malignant melanoma of the genitourinary tract is very rare and most frequently involves the distal urethra [17], with less than thirty cases reported in the literature. In addition, one must be wary of collision tumors in which a melanoma metastasizes to a primary bladder tumor. Although uncommon, there is a single case report of a melanoma metastasizing to a preexisting urothelial carcinoma [11].

In summary, to date there are 24 known cases of malignant melanoma metastatic to the urinary bladder. As “the great mimicker,” melanoma can take on various histologic appearances and it is important to keep this entity in the differential diagnosis of high grade infiltrative urothelial lesions without a papillary or in situ component. Immunohistochemical stains and a thorough clinical history and examination can provide further assurance in making the diagnosis.

## Figures and Tables

**Table 1:** Cases of malignant melanoma metastatic to urinary bladder previously reported in the English literature.

Author	Year	Age	Sex	Presenting symptom	Primary	Synchronous metastases	Treatment
Weston and Smith [10]	1964	69	M	Urinary retention	Right lower eyelid	Widespread	None

Bartone [18]	1964	70	F	Hematuria	Left thumb	Lymph nodes, brain	Partial cystectomy

Amar [19]	1964	33	M	Hematuria	Maxillary	Anterior neck	Partial cystectomy

Meyer [9]	1974	69	M	None	Unknown	Widespread	Chemotherapy

Meyer [9]	1974	60	F	Hematuria	Skin, left arm	Unknown	Surgery

Meyer [9]	1974	42	M	Incidental	Skin, right upper back	Widespread	TURBT

Silverstein et al. [20]	1974	56	M	Hematuria	Axillary lymph nodes	Absent	BCG

Tolley et al. [21]	1975	48	F	Hematuria	Vulva	Absent	Radical cystectomy

Stein and Kendall [22]	1984	50	M	Hematuria	Left arm	Absent	TURBT + chemotherapy

Irisawa et al. [23]	1987	77	M	Occult blood	Eye	Diffuse	TURBT

Arapantoni-Dadioti et al. [24]	1995	28	F	Dysuria	Axillary lymph node	Skin (abdomen, limbs), lung, and brain	TURBT

Demirkesen et al. [25]	2000	45	F	Frequency, hematuria	Right heel	Widespread	Chemotherapy

López et al. [26]	2002	75	M	Hematuria, lower urinary symptoms	Skin, scapula	Unknown	Cystectomy

Lee et al. [27]	2003	46	M	Hematuria	Skin, back	Lungs, liver, and mesenteric lymph nodes	Removed tumor and then IL-2 therapy

Fink et al. [28]	2003	38	M	Hematuria	Skin, left upper leg	Tonsils, cervical lymph nodes, esophagus, stomach, and skin	Embolization of nutrient vessels

Maeda et al. [29]	2008	65	M	Hematuria, urinary obstruction	Skin, foot	Unknown	TURBT

Nohara et al. [30]	2009	62	M	Hematuria	Skin, breast	Lymph nodes	Unknown

Efesoy and Cayan [4]	2011	60	F	Hematuria, weight loss	Skin, right middle finger	Brain, lung, and retroperitoneal and pelvic lymph nodes	TURBT

Nair et al. [6]	2011	54	M	Hematuria	Conjunctiva	Widespread	TURBT, chemo, and radiation for brain metastases

Charfi et al. [31]	2012	54	M	Unknown	Esophagus	Lumbar lymph nodes	Unknown

Paterson et al. [3]	2012	84	M	Rectal bleeding	Skin, upper back	Lungs	

Ikeda et al. [32]	2013	54	F	Hematuria	Skin, leg	Absent	TURBT

Rishi et al. [5]	2014	61	F	Dysuria, hematuria	Skin, mid-back	Brain, lung, and skin	Tumor resection, brain radiotherapy, and temozolomide

Meunier (current case)	2014	55	F	Hematuria	Skin, thigh	Liver, spleen, and lung	Ipilimumab + anti-PD-1 immunotherapy trial

## Abbreviations

IHC: Immunohistochemistry
CIS: Carcinoma in situ
TURBT: Transurethral resection of bladder tumor.
